# Supplementary material for: Causal Relationship between Aging and Anorexia Nervosa: A White-Matter-Microstructure-Mediated Mendelian Randomization Analysis
Source: Biomedicines. 2024 Aug 16;12(8):1874. doi: 10.3390/biomedicines12081874 (PMC11351342; doi:10.3390/biomedicines12081874)
Supplement: Supplementary file 1 [file biomedicines-12-01874-s001.zip › Supplementary Figure/Figure S8/Leaveoneout plot of MR analysis for TL to ubm-b-1784.pdf]

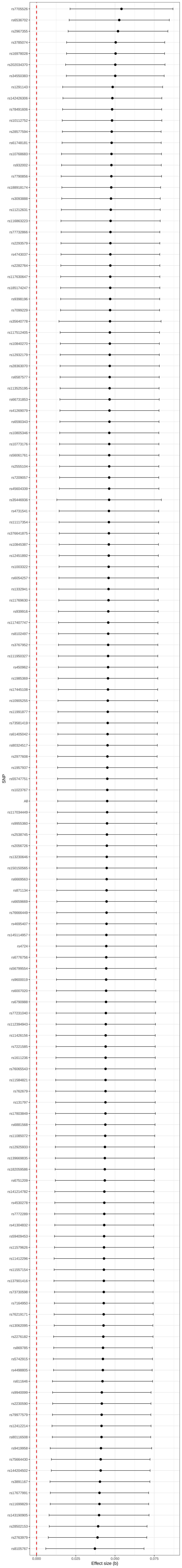

rs7705526
rs6536702
rs2967355
rs3785074
rs16978028
rs202034370
rs34550383
rs1291143
rs142426306
rs78491606
rs10112752
rs28577594
rs61748181
rs10768683
rs932002
rs7790856
rs188918174
rs3093888
rs11212631
rs116863223
rs77732866
rs2293579
rs4743037
rs2282764
rs117630647
rs185174247
rs9398196
rs7099229
rs35640778
rs117512405
rs10840270
rs12932179
rs28363070
rs6587577
rs113525195
rs66731853
rs41269079
rs6590343
rs10805346
rs10773176
rs56061761
rs2555104
rs7209057
rs45604339
rs35446936
rs4731541
rs11117354
rs376641875
rs10845387
rs12451892
rs1003322
rs6054257
rs1332941
rs11769630
rs939916
rs117407747
rs8102497
rs3767952
rs111950327
rs450962
rs1985369
rs17445108
rs10905255
rs11991877
rs73581419
rs61405042
rs80324517
rs2977608
rs1957937
rs55747751
rs1023767
All
rs117034449
rs9955360
rs2538745
rs2056726
rs13230646
rs150150565
rs6669563
rs871134
rs6659669
rs76666449
rs4695407
rs145114957
rs4724
rs6776756
rs56799554
rs9600019
rs6007020
rs6790988
rs77231040
rs112394943
rs11426156
rs7221585
rs1611236
rs76065543
rs11584821
rs762679
rs131797
rs17803849
rs6881568
rs11085072
rs12925933
rs139669835
rs182059586
rs6751209
rs141214782
rs4530278
rs7772289
rs41304832
rs59409453
rs11579626
rs11412296
rs11557154
rs137901416
rs73730598
rs7164950
rs76219171
rs13062095
rs2276182
rs869785
rs5742915
rs4498805
rs611646
rs9940099
rs2230590
rs79977579
rs12412214
rs80116508
rs9419958
rs75664430
rs144204502
rs3891167
rs17677991
rs11699829
rs143190905
rs28502153
rs2763979
rs8105767
SNP
0.000
0.025
0.050
0.075
Effect size (b)
